# Strategies which ensure our patient’s safety

**DOI:** 10.1186/2197-425X-3-S1-A76

**Published:** 2015-10-01

**Authors:** G Guillaumet, MJ Marmol, M Sola, S Guzman, L Cuenca, V Segura

**Affiliations:** Althaia Xarxa Assistencial de Manresa, Manresa, Spain

## Introduction

In 2007 Althaia carried out a project which consisted in implementing the role of the Referent Nurse. Thus, the motivation in the sanitary professional increased and the patient care improved, which brought with it the creation of a safety team in order to ensure the patients' safety. According to the Spanish Nurse Ethical Code, article 14-15 under chapter 3, all human beings are entitled to the right to life, to their safety and to the protection of their health. The nurse has the obligation to ensure and look after the patients' safety.

In our service we saw the need to set up a standardised way of monitoring procedures, records and nursing protocols.

## Objectives

To improve our patients' health.To guarantee a good implementation of healing/nursing protocols.

## Methods

Creation of a ICU safety team.Monthly meetings with the safety team.Controls of observation, intervention and records during a week every six months.Monitoring of risk indicators, effectiveness and continuity care: pain, pressure sores, check-list of aided intra-hospital transport of patients, medication errors and patient identification.

## Results

Pain: the patients showed EVE < 3 in 94,4 % of cases.

During the observation period there was a 1,76% of pressure sores91% of internal ICU transfers check-list was fulfilledMistakes when prescribing treatments: 9.4%95.5% of the patients were wearing an ID wristband.

## Conclusions

The creation of a safety team in ICU helps us to achieve our set goals. It also helps us to recognise flaws and/or improvements in our job, which has an effect on the patient's safety.

